# Fatherhood and high-risk pregnancy: a scoping review

**DOI:** 10.1186/s12884-023-05422-x

**Published:** 2023-03-16

**Authors:** Kyle Jackson, Erika Erasmus, Ntobizodwa Mabanga

**Affiliations:** Department of Psychology, University of the Western Cape, Robert Sobukwe Rd, Cape Town, 7535 South Africa

**Keywords:** Fatherhood, High-Risk Pregnancy, Masculinity, Scoping Review

## Abstract

**Background:**

The experience of expectant parenthood is commensurate of relative angst and nervousness albeit one of overall excitement and joy. However, when the pregnancy is regarded as high-risk, this experience changes dramatically for both parents. While literature on high-risk pregnancies is gaining traction, the focus is predominantly on the mother’s experiences and therefore, a paucity exists in exploring the father’s experiences of a high-risk pregnancy. This study aimed to determine the current extent of literature focusing on father’s experiences of a high-risk pregnancy using a scoping review methodology.

**Method:**

Nine databases were reviewed using the EBSCOHost metadatabase: *Academic Search Complete*; *APA PsychArticles*; *CINAHL Plus with full-text*; *Health Source: Nursing/Academic Edition*; *MasterFILE Premier*; *MasterFILE Reference eBook Collection*; *MEDLINE*; *SocINDEX with full-text*; and *eBook Collection*. Data was extracted according to the following headings: *Authors (including the year of publication)*; *aim of the study*; *research context*; *research design*; *sample characteristics*; and *key findings*.

**Results:**

Fifteen studies were included in this review. A narrative synthesis was applied within which 4 key themes emerged from the data: (1) *The father versus the healthcare professional and the hospital environment*; (2) *The impact of high-risk pregnancies on fathers*; (3) *Redefining the role of ‘father’ after experiencing high-risk pregnancy* and (4) *Focus on fathers: Recommendations for support during high-risk pregnancies*.

**Conclusion:**

The findings of this study highlights the importance of the inclusion of men and fathers in supporting both his partner and (un)born child. The findings further illustrated the long-lasting impact of trauma felt by men which constrained his ability to support his family. A family-centred approach is needed to further support the family and the impact of a high-risk pregnancy on all members within the family unit.

## Background to the study

Arguably the journey to parenthood begins immediately after a pregnancy is confirmed, with some authors suggesting that this journey commences well before conception [1]. The transition to parenthood not only incorporates great expectations but also intense worry and distress especially if the confirmed pregnancy is considered high-risk [2]. While research on mother’s experiences of high-risk pregnancy is gaining traction [3], a significant gap relates to the exploration of father’s experiences more specifically. The transition to fatherhood has a significant impact on men’s mental health and well-being and remains an important, under-researched health issue [4]. A pregnancy is considered high-risk when the life of either the mother, the foetus, or both are put at risk due to obstetric or medical complications that arise either during pregnancy, birth, or after birth [2, 5]. Obstetric complications may include gestational diabetes mellitus, pregnancy-induced hypertension, and multiple gestations, whereas medical complications may include heart disease, systemic lupus, thalassemia, and erythematosus [6].

A ‘normal pregnancy’ can have a negative psychological impact on both the mother and her partner; however, this impact is exacerbated when the pregnancy is high-risk [7]. In both instances, the focus tends to be on the impact on the mother’s well-being and less so on the father’s [8, 9]. This is particularly evident in some developed countries for example, where there are not only physical services for women that have a HRP (for pregnancy appointments; provision of food and other supplies etc.), but also mental health care services; men on the other hand do not receive such support [9, 10].

In research, fathers and fatherhood is often referenced in terms of parental status or a fertility status (which relates to being a child’s biological father). For example, some references in research are made to ‘social fathers’, which are men who take care of children who are not their biological children but through adoption or being a stepparent and/or ‘economic fathers’ which are men who adds to the maintenance of a child ([11] p29, [12] p175). In other research on fatherhood, traditional notions of masculinity seem to impede being an involved father [13]. Hegemonic constructions of masculinity dictate that fathers should assume and enact their ‘manliness’ through the dominance of others (in particular femininity and subordinate masculinities) [11, 13]. Central to this role is the ability to provide financially for his family which serves as a signifier for manhood and fatherhood [13].

A HRP is a stressful period for men as well, which means that they in turn also need support, as they are often expected to support their wives, with no one to support them [6, 9]. Typically, fathers tend to experience shock and feel isolated once their babies are born. This may be in-part due to the perception that men tend to only prepare for their paternal roles once their babies are born. This is in contrast to the perception that women prepare for their maternal role during the pregnancy as they experience both physical and emotional changes during this time [9, 14]. The transition to fatherhood becomes fragmented when he is confronted with a high-risk pregnancy (HRP). Given the lack of focus on father’s experiences of a HRP, this study aims to determine the existent literature on fatherhood within the context of a HRP and the effect thereof on father’s well-being.

## Method

In order to address the aim of the study, a scoping review methodology was determined as an appropriate research design [15]. The study utilised the 5-step guide as proposed by Arksey and O’Malley [16] as outlined in Table 1 below.

**Table 1:** Arksey & O’Malley’s [16] 5-Step Guide to conducting a scoping review

Steps Guiding the Review	Study Procedure
1. Identifying the research question	What literature exists that considers the father’s experience within the context of a high-risk pregnancy?
2. Identifying relevant studies	The search strategy included a review of the EBSCOhost metadatabase, specifically the following nine databases were screened: *Academic Search Complete; APA PsychArticles; CINAHL Plus with full-text; Health Source: Nursing/Academic Edition; MasterFILE Premier; MasterFILE Reference eBook Collection; MEDLINE; SocINDEX with full-text;* and *eBook Collection* To further guide the review the following search terms were used: “Father” OR “Dad” OR “Paternal” AND “High risk pregnancy” OR “Complicated pregnancy” OR “Medical high-risk pregnancy” OR “Birth complications” OR “Pregnancy complications” There were no limitations in terms of the time period in order to fully provide a synthesis of the landscape of available literature. However, once all Boolean phrases were included in the Ebscohost metadatabase, the search results indicated a date period of 1948–2022 The study adopted a four-pronged approach in the screening and review of the various literature. The initial search which included a review of all titles, followed by a review of all the abstracts, a review of the full-text articles against the predetermined inclusion criteria and finally a reference mining review of the included articles
3. Study selection	Throughout each stage of the review, all three authors applied the inclusion criteria to determine the appropriateness of the various titles, abstracts and full-texts in addressing the aim of the study The inclusion criteria: •Studies that focus on fatherhood and high-risk pregnancy •Studies that include both parents within the context of high-risk pregnancy (data on fathers was extracted) •Peer-reviewed studies published in English with full-text access •There were no limitations placed on the specific cause of the high-risk pregnancy •No limitations were placed on study designs The exclusion criteria: •Studies that focus on absent fathers •Studies that did not specifically focus on high-risk pregnancy •Studies that reported on mothers’ perceptions of father’s experiences
4. Charting the data	The data extraction was completed using Microsoft Excel with the following headings used as a guide: Authors; Aim of the Study; Research Context; Research Design; Sample Characteristics; Key Findings (see Table 3) Furthermore, the Preferred Reporting Items for Systematic Reviews as well as the Meta-Analyses extension for Scoping Reviews (PRISMA-ScR) was used to delineate the review process (see Fig. 1)
5. Collating, summarising and reporting the results	Narrative synthesis was used to collate, summarise and report the results of the study. This study adopted the last three steps in conducting a narrative synthesis specifically: developing a preliminary synthesis; exploring the relationships within and between studies; and finally assessing the robustness of the synthesis [17]. The second and third author conducted the synthesis as part of their thesis. This synthesis was repeated by the first author as a means of comparing and qualifying differences. These differences were discussed collectively and once an agreement was established the data was reported

### Ethics considerations

The study received ethics clearance from the University of the Western Cape’s Human Social Sciences Research Ethics Committee (HS21/5/7). All methods were carried out in accordance with relevant guidelines.

## Results

The results of the review yielded a total of 15 articles. The review process adopted a four-pronged approach which included:

1. Title review:
  2190 hits were retrieved by using the Boolean phrases above. 149 duplicates were removed and 2041 titles were reviewed against the inclusion criteria.
2. Abstract review:
  2007 articles were excluded and 34 article abstracts were reviewed of which 15 did not meet the stipulated inclusion criteria.
3. Full-text review:
  19 articles were scrutinised in the full-text review phase and 7 were excluded.
4. Reference mining:
  Finally, reference mining of all 12 articles was conducted and 2 additional articles met the inclusion criteria.
  A second round of reference mining took place on the 2 additional articles included and a third study was deemed appropriate for inclusion. This final study was also subjected to a review of the references but no additional articles were included.

Table 2 below provides an overview of the number of hits retrieved from each database.

**Table 2:** Database search results

Date of search	Keyword search	No. of publications retrieved	Database
11 February 2022	“Father” OR “Dad” OR “Paternal” AND “High risk pregnancy” OR “Complicated pregnancy” OR “Medical high-risk pregnancy” OR “Birth complications” OR “Pregnancy complications”	**896**	Academic Search Complete
11 February 2022	“Father” OR “Dad” OR “Paternal” AND “High risk pregnancy” OR “Complicated pregnancy” OR “Medical high-risk pregnancy” OR “Birth complications” OR “Pregnancy complications”	**689**	SocINDEX with Full Text
11 February 2022	“Father” OR “Dad” OR “Paternal” AND “High risk pregnancy” OR “Complicated pregnancy” OR “Medical high-risk pregnancy” OR “Birth complications” OR “Pregnancy complications”	**383**	CINAHL Plus with Full Text
11 February 2022	“Father” OR “Dad” OR “Paternal” AND “High risk pregnancy” OR “Complicated pregnancy” OR “Medical high-risk pregnancy” OR “Birth complications” OR “Pregnancy complications”	**222**	APA PsycArticles

### Characteristics of the included studies

**Fig. 1:**
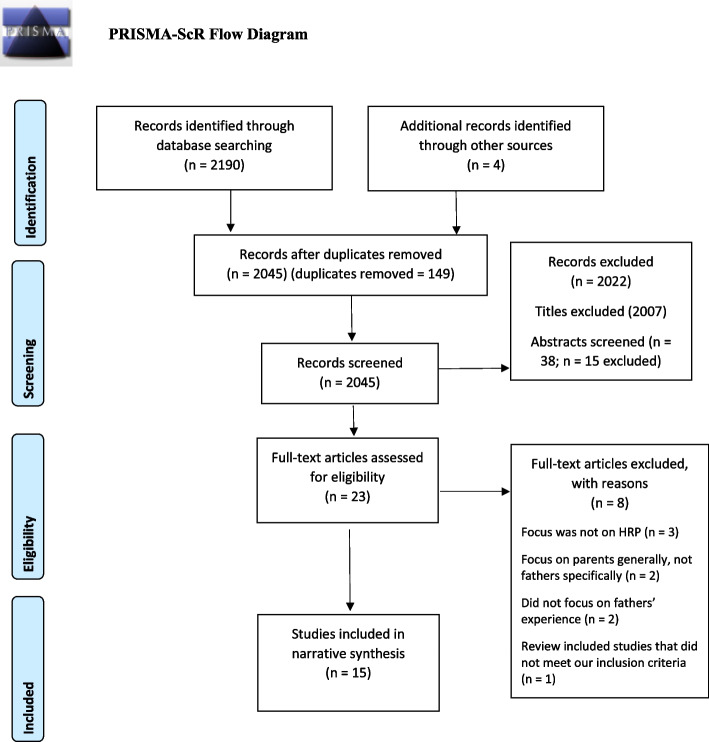
PRISMA-ScR Flow Diagram [32]

Of the 15 included studies, 11 were from High-Income countries and 4 were from Low-Middle Income countries. In particular, 4 were reported from the United States, 3 from Sweden, 2 from Uganda and the United Kingdom and 1 from Germany, Taiwan, Malawi and Thailand. The research designs for the 15 studies included a secondary data analysis of individual interviews [2]; cross-sectional survey [1], a retrospective chart review [1] and individual interviews [11] of which one study also included a focus group discussion. Table 3 provides more details concerning the data extraction. Figure 1 below provides a summary of the search process using the PRISMA-ScR diagram.

**Table 3:** Data extraction

	**Authors**	**Year**	**Country**	**Research Aim**	**Research Design**	**Data Collection Methods**	**Sample Characteristics**	**Related Themes**
1	Koppel & Kaiser [18]	2001	Germany	To examine the situation of fathers with a newborn child on an intensive care unit	Qualitative approach	In-depth individual interviews	18 Fathers in NICU	The father versus the healthcare professional and the hospital environment The impact of high-risk pregnancies on fathers Redefining the role of ‘father’ after experiencing high-risk pregnancy Focus on fathers: Recommendations for support during high-risk pregnancies
2	Moore et al. [19]	2019	United Kingdom	To explore the discursive construction and social actions achieved by accounts given by men following a birth in which the mother developed life-threatening complications	Qualitative approach	Individual interviews, Secondary data analysis	4 Fathers	The father versus the healthcare professional and the hospital environment The impact of high-risk pregnancies on fathers
3	McCain & Deatrick [20]	1994	Ohio, America	To describe the experience of high-risk pregnancy from the perspectives of mothers and fathers	Qualitative approach	Individual interviews, Secondary data analysis	21 Parents (12 mothers and 9 fathers)	The impact of high-risk pregnancies on fathers Redefining the role of ‘father’ after experiencing high-risk pregnancy
4	Nansubuga & Ayiga [21]	2015	Rakai District, Central Uganda	The study examined the roles played by men after the onset of maternal near miss complications in Uganda	Both qualitative and quantitative (retrospective, cross-sectional study)	Narratives and in-depth individual interviews	40 maternal near-misses and 10 partners	Redefining the role of ‘father’ after experiencing high-risk pregnancy
5	Hsieh et al. [22]	2006	Southern Taiwan	To evaluate the experiences of first-time expectant fathers with a tocolyzed spouse	Qualitative approach: Descriptive phenomenological design	In-depth individual interviews	6 first-time fathers	The father versus the healthcare professional and the hospital environment The impact of high-risk pregnancies on fathers Focus on fathers: Recommendations for support during high-risk pregnancies
6	Aarnio et al. [23]	2018	Mangochi district, Malawi	To provide information about husbands’ role in decision-making and healthcare seeking in cases of pregnancy complications	A qualitative interview study	In-depth individual interviews	24 individuals, with 12 of them being the fathers and the other 12 the mothers	The father versus the healthcare professional and the hospital environment Redefining the role of ‘father’ after experiencing high-risk pregnancy Focus on fathers: Recommendations for support during high-risk pregnancies
7	Cole et al. [24]	2016	Philadelphia, Pennsylvania	To describe the incidence of psychological distress (symptoms of post-traumatic stress and de pression as endorsed on objective measures) among expectant parents, shortly after they received the diagnostic confirmation of a fetal anomaly at a high-risk fetal centre	A 2-year retrospective medical chart review	CFDT mental health screening tool and the Revised Impact of Events Scale (IES-R)	1820 participants were screened, with 788 being expectant fathers and 1032 expectant mothers	The impact of high-risk pregnancies on fathers
8	Linberg & Engström [25]	2013	Sweden	The objective of the study was to describe new fathers’ experiences of care in relation to complicated childbirth	A qualitative thematic content analysis	In-depth individual interviews	8 fathers	The father versus the healthcare professional and the hospital environment The impact of high-risk pregnancies on fathers Focus on fathers: Recommendations for support during high-risk pregnancies
9	Maloni & Ponder [26]	1997	United States	To describe the problems and stress of men whose pregnant partners are on bed rest and the assistance they received	Cross-sectional survey design (descriptive retrospective approach)	The Paternal Bed Rest Questionnaire (PBRQ) of open-ended questions	59 Caucasian men of partners with prescribed antepartum bed rest	The impact of high-risk pregnancies on fathers Redefining the role of ‘father’ after experiencing high-risk pregnancy Focus on fathers: Recommendations for support during high-risk pregnancies
10	Patel et al. [27]	2018	Sweden	To explore the experiences of healthcare in fathers whose partner was suffering from peripartum cardiomyopathy	Qualitative research design	In-depth individual interviews	14 fathers of which 8 are first-time fathers with partners presenting symptoms of peripartum cardiomyopathy (PPCM)	The father versus the healthcare professional and the hospital environment The impact of high-risk pregnancies on fathers Redefining the role of ‘father’ after experiencing high-risk pregnancy Focus on fathers: Recommendations for support during high-risk pregnancies
11	Patel et al. [28]	2019	Sweden	To learn more about fathers’ reactions over their partner’s diagnosis of peripartum cardiomyopathy	Qualitative research design	In-depth individual interviews	14 fathers of partners with (PPCM)	The impact of high-risk pregnancies on fathers Redefining the role of ‘father’ after experiencing high-risk pregnancy Focus on fathers: Recommendations for support during high-risk pregnancies
12	Tanasirijiranont et al. [6]	2019	Northern Thailand	The research question was: “What is going on in the process of becoming a first-time father among Thais whose wives have a high-risk pregnancy?”	Grounded Theory design	In-depth individual interviews	23 Thai men informants	The impact of high-risk pregnancies on fathers Redefining the role of ‘father’ after experiencing high-risk pregnancy Focus on fathers: Recommendations for support during high-risk pregnancies
13	May [29]	1994	United States; Southern State	To describe the impact on expectant fathers of their partners' activity-restricted pregnancies	Qualitative	Phase 1: semi-structured interview Phase 2: semi-structured focus group discussion	Phase 1: 15 Fathers 2 weeks after partners restriction and Phase 2: 15 fathers 1–2 years after partners activity-restricted pregnancies	The father versus the healthcare professional and the hospital environment The impact of high-risk pregnancies on fathers Redefining the role of ‘father’ after experiencing high-risk pregnancy
14	Kaye et al. [30]	2014	Uganda	To gain a deeper understanding of their experiences of male involvement in their partners' healthcare during pregnancy and childbirth	Qualitative	In-depth individual interviews	16 Fathers whose wives were admitted to hospital for severe obstetric complications	The father versus the healthcare professional and the hospital environment The impact of high-risk pregnancies on fathers Focus on fathers: Recommendations for support during high-risk pregnancies
15	Hinton et al. [31]	2014	UK	To explore the impact of near-miss obstetric emergency, focusing particularly on partners	Qualitative	In-depth individual interviews	35 women, 10 male partners and 1 lesbian partner	The father versus the healthcare professional and the hospital environment The impact of high-risk pregnancies on fathers Focus on fathers: Recommendations for support during high-risk pregnancies

### Narrative synthesis

The study adopted the narrative synthesis framework as suggested by Popay et al. (2006), in particular the last 3-steps. Specifically, the steps involved were to (1) develop a preliminary synthesis by arranging the findings of the various studies to explore the patterns across each; (2) explore the relationships in the data to be able to explain the patterns/discrepancies that were noted across the various included studies and finally; (3) to assess the robustness of the synthesis whereby the authors specifically reviewed the various patterns and discrepancies to determine possible explanations and conclusions from the emergent data of the included studies. This was further supported by generalising conclusions to other populations and contexts as evidenced by the population samples of the included studies [17].

The results of the included articles are synthesized into the following 4 themes: (1) *The father versus the healthcare professional and the hospital environment*; (2) *The impact of high-risk pregnancies on fathers*; (3) *Redefining the role of ‘father’ after experiencing high-risk pregnancy* and (4) *Focus on fathers: Recommendations for support during high-risk pregnancies.*

### The father versus the healthcare professional and the hospital environment

The first major theme that emerged from the data is concerned with father’s interactions with healthcare staff and navigating an environment in which they may not always feel welcome.

In terms of father’s interactions with healthcare staff, fathers reported a lack of communication, feelings of neglect and near-total exclusion from healthcare professionals concerning issues of their spouses. The lack of communication experienced by fathers by healthcare professionals often lead to father’s inability to adequately prepare for worse-case-scenarios, which increased their feelings of alienation and insecurity and precipitated feelings of anxiety and powerlessness [19, 25, 30]. As such, fathers indicated that they did not have a lot of knowledge regarding the prognosis of the high-risk pregnancy and its associated complications. This information was often given to mothers by healthcare professionals which impedes the father’s ability to assist the mother when complications arise [23]. This exclusion can lead to role ambiguity and not knowing what was going to happen to their partners or their child [25]. These negative experiences are therefore related to fathers’ being at the periphery and a lack of acknowledgement by healthcare professionals as well as being related to inadequate healthcare received by their partners [22, 27]. This lack of support was also extended beyond the birth through to follow-up assessments and outpatient care in which fathers experienced neglect from nursing staff [29].

Fathers characterised the hospital environment as unwelcoming. This was exacerbated by a lack of privacy and an absence of facilities and space for men to occupy within the hospital environment, which is not uncommon within low-income settings [30]. Fathers expressed that they wanted to be involved and respected [27]. In one study, fathers were excluded from being present during the birth of their child without receiving adequate information as to why they were excluded [18]. Contrastingly, in the study by Hinton et al. [31] participants from a high-income country indicated that they were surprised and shocked to experience life-saving interventions for high-risk pregnancies particularly when they considered childbirth as a safe and routine procedure.

A high-risk pregnancy can be considered a traumatic experience for both partners. The lack of acknowledgement from healthcare professionals in recognising the trauma faced by men impedes the help-seeking behaviours of men specifically within the context of hegemonic masculinity [19].

### The impact of high-risk pregnancies on fathers

The second major theme centred on the impact and effects of the high-risk pregnancy on fathers specifically.

There was a consistent reaction experienced by fathers, as reported in most studies, those were, fathers feeling extremely stressed and near total exhaustion of mental resources [18, 20, 22]. Stress was largely related to maintaining employment, ensuring that household tasks were still completed and ensuring that the mother felt supported [29]. Supporting the mother and infant served as the primary focus for fathers, often at the expense of their own need for support so as to not detract from the needs of their partners [27]. Fathers attempted to ensure that mothers felt supported by hiding or controlling their emotions and fears from their partners [26]. To outwardly demonstrate being strong and secure was essential to fathers as a sign that they were able to cope and an admission of fear was viewed as a sign of weakness [28]. The experience of a high-risk pregnancy was perceived as a period of extreme pressure, concern, worry and confusion [6]. Fathers expressed feeling torn between wanting to be there for their partners and wanting to be there for their babies [25].

As a result, fathers are at an increased risk for the onset of mental health problems which is further exacerbated by the reorganisation of the self (serving as both the breadwinner and ‘single’ parent) often without support [28]. Cole et al. [24] determined that 14% of fathers within their study were at risk of developing Major Depressive Disorder (MDD). They also determined that 8.1% of fathers had elevated symptoms of both traumatic stress and depression. These findings are contrasted with the findings of May [29] where 30% of fathers in that study reported clinical levels of depression.

Some of the effects experienced by fathers were as a result of their experiences with healthcare professionals and in the hospital setting. Although support and communication are valued, it is often lacking and the long-term mental health consequences are noted [31]. The hardest experience for fathers was to witness their partners in pain and to not be able to intervene.

Childbirth, in particular high-risk pregnancies, can be traumatic for fathers leaving them in a state of vulnerability and fearful for their partner and child. The father is cast aside into a position of powerlessness, conflicted and in limbo as a spectator [27]. The impact of this predisposes men to psychological and mental scarring which is attributed to being alienated, ignored or mistreated by healthcare providers [30]. These experiences further contributed to men’s feelings of fear and anxiety which culminated into profound long-term consequences for both men and their partners [31].

The impact for fathers is also experienced at home, when mothers are placed on bedrest or activity restriction for the remainder of the pregnancy which results in household disruption. In the study by May [29], the effects of maternal activity restriction resulted in accidental cases of child poisoning, maternal postpartum depression (which in turn, impacts the father), child abuse and significant marital strain.

Beyond this, guilt was also experienced by fathers as they thought they had a better experience in comparison to their partners [25]. In an effort to cope, fathers employed a strategy of cognitive refraining in changing their focus from themselves to that of the mother of the foetus and calculating cost–benefit ratios [26]. Furthermore, men indicated that they felt inspired by their unborn child to be brave and a desire to exchange their lives for the lives of their baby [6]. By being involved, fathers felt more connected and this strengthened the relationship between fathers and their partners [25].

Ultimately, men need to negotiate the dilemma of maintaining traditional constructions of masculinity within the context of disempowerment, lack of agency and confrontation with their own vulnerability [19]. These narratives call on men to reconstruct their identities and how they imagine their future. Within this, family relationships serve as the primary source for healing and shared-meaning making [19]. Although this period is incredibly difficult for both parents, fathers indicated that it was worthwhile particularly if the mother and child came through healthily [26, 29].

### Redefining the role of ‘father’ after experiencing high-risk pregnancy

The next theme to arise from the data, is focused on fathers reconstructing their ideas of what it means to be a father in relation to the experience of a high-risk pregnancy.

As determined in the previous theme, fathers’ reactions to a high-risk pregnancy was characterised as being fearful for their wives and once fathers knew that their wives were safe, their fear turned to their child [18]. Furthermore, fathers sought to provide support for their wives at the expense of their own needs [20]. Moreover, in the study conducted by Aarnio et al. [23] they found that fathers regretted not being part of the pregnancy experience when they were side-lined, and not receiving updates regarding the status of their wives or child(ren).

Interestingly, hegemonic masculinity seems to be a predominant theme amongst fathers in the included studies, albeit to varying degrees. Childbirth can be traumatic for fathers who want to remain strong in the face of their vulnerability and fear for the partner and infant. This façade hinders fathers from seeking professional support [28]. This was also supported by Aarnio et al. [23] in which fathers are required to take care of their wives and if they are unable to do so, their social capital is directly influenced. In a short space of time, a father is required to re-evaluate his role as he tries to support, care and stand-up for his family [27].

The roles that fathers undertake during this time include not only the assumed duties and responsibilities of each parent but to also execute these roles well which may include: being a father; a good husband/partner; a strong family leader; a son; active member of society and a worker. Coupled with this he is also planning for any uncertainties concerning the care for the mother, baby, the financial and work implications thereof [6, 29]. The stress associated with caring for their partners, maintaining the family’s financial status and their own mental health was pervasive and all encompassing. This increased burnout, depression and placed significant strain on the marital relationship between the father and the mother [26].

According to Tanasirijiranont et al. [6] a core theme within their study was fathers’ concern for the well-being and health of their unborn child. This translated into striving for increased care (nutrition, medicine, dietary control) in the hope that the high-risk pregnancy condition would not become worse.

In other instances, fathers assume the role of heads of the household and therefore, they are typically the one’s left to make decisions that impact on the economic resources of the family. These include which hospital to attend and how to manage any pregnancy complications that may arise [23]. In preventing maternal deaths and reducing the risk of pregnancy, the study by Nansubuga and Ayiga [21] highlight the role played by men. Firstly, this included the long-term or permanent use of contraceptives. Secondly, the study highlighted the roles of fathers in averting maternal death by managing obstetric complications within the household (e.g. administering essential haemorrhage medication (oral or injectable), massaging the uterus when the placenta was not expelled). Finally, the study highlighted the supportive roles undertaken by men including decision-making during emergencies, financial support and access to obstetric care, transport, social and emotional support etc. Fathers in this study believed that they were financially obligated to ensure that their wives had access to emergency medical services even if it meant borrowing or selling assets to achieve this. In terms of support, fathers sought to accompany their wives to healthcare facilities, providing emotional support and caring for sick mothers. This study highlights the importance of the role of the father in being engaged in birth preparedness and complication readiness [21].

### Focus on fathers: Recommendations for support during high-risk pregnancies

The final theme that emerged from the synthesis details the support needed and recommendations made by fathers in navigating the context of a high-risk pregnancy.

Central to the essence of fathers’ experiences of high-risk pregnancy was their interactions between the hospital environment and healthcare practitioners. Expectant fathers adopt the role of central caregiver, increased concern over a shortage of income and time which increases the pressure he faces [22]. Fathers stressed the importance of improved communication and support from nurses and physicians [18, 25], which culminates in fathers feeling a sense of control and facilitates a greater sense of coping [28]. Feeling prepared and receiving clear, honest communication were essential elements to an overall positive experience which enables fathers to provide better support to their partners [27].

This was demonstrated further in the study by Lindberg and Engström [25] where fathers appreciated status updates on their partners and babies and being invited to the c-section (whether inside or outside the operating room) as it validated fathers’ presence and desire to support his family. In the study by Patel et al. [27] only one (*n* = 14) father reported an instance where the family was invited to receive a status update on the wife and child which highlights the lack of family centredness. Family care tends to exclude the father which may impede further involvement and contravenes the major role he plays in supporting his partner [26]. Nursing staff should provide proper assistance and support for fathers, never excluding him from the nursing service [22].

In the study by Tanasirijiranont et al. [6], fathers relied on their faith to guide them through their experience. This was related specifically to the Buddhist concept of karma and therefore, in a way to improve the status of their wife and baby, fathers engaged or did not engage in various activities or tasks as a means to try and increase good karma and protect his wife and expectant child.

The study by Hinton et al. [31] highlighted that the interview was often fathers’ first opportunity to talk about their experiences, with some interviews taking place years after the experience. This demonstrates the pervasive long-lasting trauma experienced by fathers and a sense of isolation owing to the ‘rarity’ of their experience. Fathers indicated that they would like stronger male networks that can help them with their wives’ pregnancy complications and overall improved maternal health knowledge for husbands [23]. Interventions to improve male involvement should focus on educational support, a conducive environment, motivational information and positive healthcare provides attitudes towards fathers [30].

It is therefore noteworthy that despite the existence of supportive policies (in some contexts) for male involvement, men’s experiences highlight the dissonance between social expectations and men’s experiences as well as the dissonance between policy for male involvement and practice within the health system [30].

## Discussion

This study aimed to provide a synthesis of the current landscape of literature that reports on fathers’ experiences of high-risk pregnancy. The results of the synthesis demonstrated a range of experiences, which was synthesised into 4 themes: (1) *The father versus the healthcare professional and the hospital* environment; (2) *The impact of high-risk pregnancies on fathers;* (3) *Redefining the role of ‘father’ after experiencing high-risk* pregnancy; and (4) *Focus on fathers: recommendations for support during high-risk pregnancies*.

The first theme focused on the lack of communication, the experience of neglect and near-total exclusion from healthcare staff. This increased fathers’ sense of alienation and insecurity which exacerbated their anxiety, sense of powerlessness and role ambiguity in not knowing how to be there to support their partner and child [19, 25, 30]. The exclusion of men was experienced both in terms of *institutional* exclusion (lack of privacy, absence of facilities or spaces for men to occupy within the hospital environment) as well as amongst healthcare *professionals* (lack of support, information and acknowledgement of his role and trauma experienced). This ultimately impacted the help-seeking behaviour of fathers [18, 19, 22, 23, 27, 29, 30].

The second theme indicated that fathers sought to support their partners and be involved in the pregnancy journey which culminated in adopting various, conflicting roles and responsibilities. These included maintaining all their employment responsibilities; managing the household effectively (the re-organising of the self by simultaneously assuming both the roles of primary caregiver and breadwinner) as well as maintaining his primary focus on supporting his partner and child at the expense of his own needs, leading to near total exhaustion of mental resources [18, 20, 22]. In many of the studies, showing emotion was considered a weakness and therefore fathers sought to outwardly demonstrate being strong and secure [26–29]. The dissonance of what is felt internally versus what is expressed outwardly often increases his predisposition to psychopathology, specifically major depressive disorder [24, 28, 29]. The trauma of the high-risk pregnancy, neglect of his own needs, exhaustion of maintaining the various roles, his reorganisation of self, anxiety, fear, guilt, burnout, lack of support and being cast aside imprints mental scarring with long-lasting effects [24, 27–29]. This was captured by fathers in explaining that the most challenging experience was to witness their partners in pain and not being able to intervene [30, 31]. The position of powerlessness conflates fathers state of vulnerability and fear for their partner and child [6, 19, 25].

The third theme dovetails the second theme in terms of a reorganisation of self. Hegemonic masculinity seemed to be a central concept amongst fathers [6, 19–21, 23, 27–29]. This often manifested in fathers wanting to show strength, neglect his own needs, wanting to support, provide for and protect his partner and (un)born child [18, 20, 23, 27, 28]. However, some studies highlighted the importance of the role of the father as primary figures of support for mothers and the impact of his role on the mother [6, 26, 29]. These behaviours and expectations increased symptoms of burnout and depression among fathers and impeded help-seeking behaviours. The birth preparedness and complication readiness of fathers served as a key factor in minimising maternal death [21]. This translated into increased care (nutrition, dietary control; medication etc.) for the well-being of mothers and the unborn child as a means to minimise the impact associated with a high-risk pregnancy [6].

The final theme demonstrated fathers’ call for improved communication and support within the healthcare system. This was seen as important since it has the potential to increase their felt sense of control and coping [18, 25], validate his presence positively which impacts on the support received by the mother [28]. This finding demonstrates the importance of incorporating a family-centred approach amongst parents experiencing a high-risk pregnancy [27]. This further converges the dissonance between social expectations, the experiences of men, the policy for male involvement and the practice felt within the healthcare system [22, 26, 30]. The rarity of the ‘father’ experience was also highlighted with the individual interviews serving as the first opportunity for men to talk about their experiences, in some cases years post the pregnancy highlighting the long-lasting trauma and isolation experienced by fathers [23, 30, 31].

## Conclusion

The study’s findings highlight the importance of focusing on the inclusion of men and fathers in the healthcare of partners and (un)born children by both healthcare staff and healthcare facilities. The involvement of men and fathers has a direct impact on the mother and thereby an indirect influence on the child. The impact of a high-risk pregnancy on the father tends to have long-lasting effects which constrains his ability to be able to sufficiently maintain the various roles he is expected to fulfil in support of his partner and child. Incorporating a family centred approach by including the role of the father, thereby validates his presence and allows for a greater felt sense of contributing and facilitating the health of his partner and child.

Future research should evaluate various policies and legislature guiding healthcare facilities and the involvement of the father as it translates into physical spaces and hospital governance. Interventions should seek to incorporate aspects that provide fathers with assistance and guidance beyond the ‘typical’ birth thereby increasing aspects of birth preparedness and complication readiness. Support services for fathers within the hospital environment should consider the multifaceted impact of a high-risk pregnancy on fathers and this awareness should be extended to post-natal check-ins and assessments. Considering the elements of hegemonic masculinity within the findings of the studies, future research should examine the contextual and cultural influence of the fathers’ experience as it relates to a high-risk pregnancy and the impact thereof on his sense of masculinity and fatherhood.

This study sought to explore the current literature base on fathers’ unique perspectives and experiences of a high-risk pregnancy through a scoping review design. This design allowed for a synthesis of the findings and to establish gaps within the literature for future research. The study was limited in terms of the number of databases searched and the published material that was included and therefore, seminal findings from unpublished sources may have been omitted. Most of the included studies were qualitative and therefore, data on the trends of fathers’ experiences is limited. The study was also limited in terms of its particular focus on the experience of fathers whilst not including studies that may have incorporated both the mother and fathers experience. In addition, other key stakeholders such as healthcare professionals etc. were not included in this review which may detract from the information obtained.

## Data Availability

All data generated or analysed during this study are included in this published article and its supplementary information files (specifically Table 3).

## Abbreviations

HRP: High-Risk Pregnancy
PRISMA-ScR: The PRISMA extension of scoping reviews
